# How Can Insulin Resistance Cause Alzheimer’s Disease?

**DOI:** 10.3390/ijms24043506

**Published:** 2023-02-09

**Authors:** Ji Hye Yoon, JooHyun Hwang, Sung Un Son, Junhyuk Choi, Seung-Won You, Hyunwoo Park, Seung-Yun Cha, Sungho Maeng

**Affiliations:** 1 Age-Tech Service Convergence Major, Graduate School of East–West Medical Science, Kyung Hee University, Yongin-si 17104, Republic of Korea; 2 Department of Comprehensive Health Science, Graduate School of East–West Medical Science, Kyung Hee University, Yongin-si 17104, Republic of Korea; 3 Health Park Co., Ltd., Seoul 02447, Republic of Korea

**Keywords:** dementia, amyloid beta, hypometabolism

## Abstract

Alzheimer’s disease (AD) is a neurodegenerative disorder associated with cognitive decline. Despite worldwide efforts to find a cure, no proper treatment has been developed yet, and the only effective countermeasure is to prevent the disease progression by early diagnosis. The reason why new drug candidates fail to show therapeutic effects in clinical studies may be due to misunderstanding the cause of AD. Regarding the cause of AD, the most widely known is the amyloid cascade hypothesis, in which the deposition of amyloid beta and hyperphosphorylated tau is the cause. However, many new hypotheses were suggested. Among them, based on preclinical and clinical evidence supporting a connection between AD and diabetes, insulin resistance has been pointed out as an important factor in the development of AD. Therefore, by reviewing the pathophysiological background of brain metabolic insufficiency and insulin insufficiency leading to AD pathology, we will discuss how can insulin resistance cause AD.

## 1. Introduction

Sir Harold Himsworth suggested the relationship between insulin resistance and CNS function more than 80 years ago [1]. He said that brain aging is associated with reduced insulin effectiveness, and that it is caused by a decrease in insulin reactivity in brain cells or a decrease in the amount of insulin passing through the blood–brain barrier (BBB). In other words, age-related brain insulin abnormalities are one of the causes of cognitive decline in MCI (mild cognitive impairment) and AD (Alzheimer’s disease).

Dementia is a neuropsychiatric syndrome in which cognitive function declines due to degenerative changes in nerve cells. As aging is a known major risk factor for dementia, and in an aging society dementia has emerged as a main health problem [2]. Currently, there is no apparent cure for dementia, and early diagnosis with prophylactic treatment to delay the disease progression is the only possible countermeasure [3]. Drugs used in clinical practice only provide temporary relief from clinical symptoms, but do not prevent the progression of the disease [4]. Accumulation of Aβ (amyloid beta) and p-tau (phosphorylated tau) in the brain is known as a pathological factor that causes neuronal death in AD, but novel drug candidates that remove these proteins have not proven their effectiveness in clinical trials [5]. Accordingly, it is necessary to develop a method to prevent the progression to AD by understanding and removing the preceding factors causing the accumulation of Aβ and hyperphosphorylation of p-tau.

Currently, the ‘amyloid cascade theory’ is losing its persuasive power, and AD is revealed to be a syndrome caused by various pathophysiological mechanisms [6]. For example, Aβ plaque is not found in one-third of patients diagnosed with AD, and cognitive impairment is often not observed even when pathological findings of AD appear in brain tissue [7]. In addition, it is very difficult to predict the progression of AD, as the cognitive impairment can progress slowly or rapidly, many symptom subtypes mainly affect either executive functions, cortical visual or swallowing, and there are psychosis- and depression-associated types, and metabolic dysfunction-associated types such as insulin resistance, hormonal deficiency, homocysteinemia [8,9,10].

Among them, insulin resistance is a factor that has received vast attention. There are many pieces of evidence suggesting that the glucose metabolism in the brain decreases more than 10 years before the occurrence of dementia symptoms [11]. So, it is presumed that the change in metabolism in the brain tissue is closely related to the onset of AD [12]. In addition, age-related dementia shares enough characteristics with insulin resistant diabetes that it is referred to as type 3 diabetes [13]. Therefore, the relevance of insulin resistance in the pathogenesis of AD will be discussed. In this review, we will address the pathophysiology of AD, evidence that AD is associated with insulin resistance, the process by which insulin resistance develops and its consequences in the brain, and the processes that lead to dementia.

## 2. Definition and Epidemiology of AD

Dementia is a complex of neuropsychiatric symptoms in which cognitive function progressively declines due to degenerative changes in brain tissue. More than 55 million people worldwide suffer from dementia, and 10 million new patients emerge every year [14]. Currently the physical, mental, and socioeconomic impacts for patients and caregivers are so great that the social burden cost USD1.3 trillion worldwide in 2019 and is expected to be 2.8 trillion in 2030 [14].

Dementia is classified into a few types depending on the cause. Among them, AD is the most common and comprises 60–70% of dementia cases [15]. Other types include vascular dementia, dementia with Lewy bodies, frontotemporal dementia, etc. Stroke, HIV infection, alcoholism, traumatic brain injury, and malnutrition are antecedent conditions that cause secondary dementia [16].

By the severity of symptoms, AD is divided into early, intermediate, and late stages [17]. Early-stage symptoms are forgetfulness and loss of orientation to time and place. In the middle stage, people often forget recent events or names of people around them, have difficulty communicating, and often need help from others. Behavior changes such as moving around or asking repetitive questions also appear. At the late stage, patients have difficulty in normal life and need care from others due to severe memory impairment, gait disturbance, aggression, and loss of orientation of time and space [14].

According to the pharmacologic effect, FDA-approved AD drugs are classified into symptom relievers and disease progression inhibitors [18]. Drugs for symptom relief are acetylcholinesterase inhibitors, NMDA(N-methyl-D-aspartate) receptor antagonist and orexin receptor blocker. Many disease progression inhibitors such as Aβ eliminators are under trial; therefore, more clinical data are needed to prove its effectiveness. As such, compared to the rapid increase in the number of patients with AD, novel drug development is progressing slowly.

## 3. Pathophysiology of AD

AD is characterized by a senile plaque in which Aβ is aggregated between brain cells and by a neurofibrillary tangle in which hyperphosphorylated tau is aggregated in nerve cells, resulting in the pathological process [19]. Aβ is produced by the cleavage of amyloid precursor protein (APP) in the cell membrane by β- and γ-secretases. Tau is a protein composed of microtubules involved in intracellular skeleton and material movement. When tau protein is hyperphosphorylated, tau entangles with other tau, and the microtubule structure destabilizes [20].

### 3.1. Functions of APP and Tau

**Figure 1:**
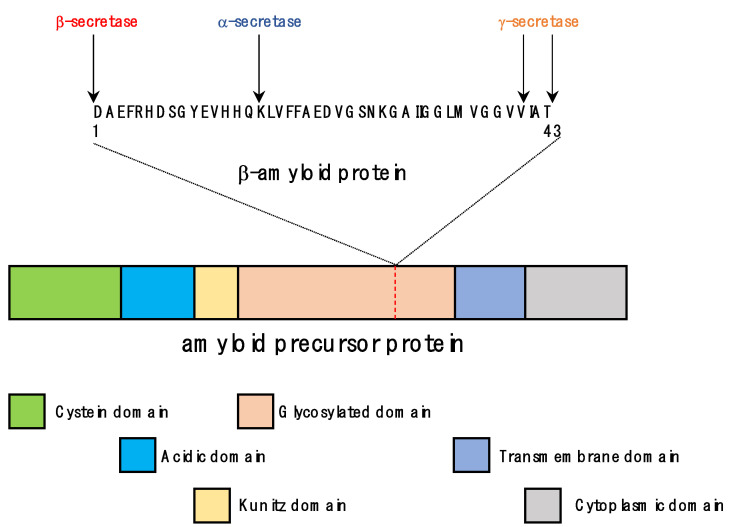
Domains and secretase sites of action of the APP (Amyloid precursor protein). APP is a transmembrane protein composed of 6 domains. From the N-terminal region these domains include a cystein domain, an acidic domain, kunitz-type protease inhibitor domain (not present in APP_695_), a glcosylated domain and a cytoplasmic domain. The glycosylated domain contains the amyloid beta sequence cleaved by α-, β-, and γ-secretase.

Human *APP* gene was identified on chromosome 21 in 1987 as a gene of the APLP (APP-related protein) family along with APLP1 and APLP2 [21]. More than 25 mutations have been found to be related to hereditary AD (familial AD) [22]. APP is a transmembrane protein and has three isoforms: APP_695_, APP_751_, and APP_770_, and among them APP_695_ is most common in neuronal cells [23]. APP is composed of a large extracellular domain, one membrane spanning (penetrating) domain, and a cytoplasmic domain (Figure 1). Although the function of APP has not yet been clearly elucidated, it is known to bind metals (copper, zinc) and extracellular matrix molecules (heparin, collagen, laminin), acts as a neurotrophic factor, provides a binding site for cell adhesion factors, inhibits proteolytic enzymes, etc. [24]. As a neurotrophic factor, APP stimulated the proliferation of fibroblasts and improved memory by increasing the number of synapses in animal experiments [25,26]. Memory improving effect was also exhibited by fragments of APP cleaved by α-secretase [27]. In addition, it was suggested that the RHDS motif of APP acts as a cell adhesion factor like an integrin, and may also serve as a receptor for F-spondin, which has regulatory function of neuronal development and damage repair [28,29]. As such, APP is presumed to be an important substance with various functions. However, APP-lacking mice showed no significant external abnormalities except for slightly reduced body weight and weaker muscle strength in the extremities. Additionally, there was no significant difference in brain cells except for nonspecific proliferation of astrocytes [30]. However, mice in which *APP* and *APLPs* genes were removed died immediately after birth, suggesting that APP-family genes compensate in the development of the nervous system [31]. 

APP is synthesized in the endoplasmic reticulum and undergoes various post-translational modifications such as N- and O-glycosylation, ectodomain and cytoplasmic phosphorylation, and tyrosine sulfation [32]. Less than 10% of initially synthesized APP reaches the cell membrane; most of the rest stays in the Golgi and TGN (trans-Golgi network) [32]. In non-neuronal cells, the APP that reaches the cell membrane is endocytosed again within a few minutes and enters the endosome. From there, some moves back to the cell membrane, and the rest goes to the lysosome and is degraded. APP is cleaved by α-secretase when APP is in the cell membrane, and by β-secretase when APP stays in the endosome or TGN [33].

The APP cleaving secretase enzyme cuts and removes APP, and there are three secretase types, α, β, and γ, that act on different cleavage positions [34]. α-secretase corresponds to zinc metalloproteinases such as TACE/ADAM17, ADAM9, ADAM10, and MDC-9 and aspartyl proteases such as BACE2 (β-site APP-cleaving enzyme 2). It is distributed near the cell membrane and cleaves between Lys^16^ and Leu^17^ in the Aβ domain of APP [35,36]. Among the β-secretases, BACE1 is a transmembrane aspartyl protease, mainly distributed in the Golgi/TGN and endosomes. It excises the N-terminus of the Aβ domain in the APP [37,38]. γ-secretase is composed of four subunits, presenilin-1 or -2, nicastrin, APH-1, and PEN-2, and is distributed in many parts of the cell such as the endoplasmic reticulum, Golgi, TGN, endosome, and cell membrane. It can cut several regions of the transmembrane domain of APP, so various sizes of Aβ peptides can be produced depending on the cut position [39]. Most of the Aβ peptides are 38 to 43 amino acids in length. A total of 90% of them are Aβ_40_ composed of 40 amino acids, and less than 10% are Aβ_42_ composed of 42 amino acids, which is more toxic than Aβ_40_ [40].

Early onset AD is associated with a genetic predisposition increasing the Aβ_42_/Aβ_40_ ratio [41]. However, early-onset AD accounts for only 5% of all AD cases, and late-onset AD is mainly caused by a combination of genetic and environmental factors. These factors causing homeostatic changes, such as an increase in the production and aggregation of Aβ, decrease in the removal and degradation of Aβ, inflammation, and a change in γ-secretase activity are related with the pathological process of AD. However, APP, PS1 and Cdk5 (cyclin-dependent kinase 5) are also associated with neuronal cell death [42,43]. In addition, tau and ApoE4 (apolipoprotein E4) can induce synaptic dysfunction and neurodegeneration, leading to AD [44].

Microtubule is cytoskeletal protein which supports the cellular structure. Microtubule is composed with 90% tubulin, and the remaining 10% is MAPs (microtubule-associated proteins) which are MAP1, MAP2 and tau protein [45]. Tau was first discovered by the Kirshchner’s laboratory in 1975 and has attracted attention as a caustic substance of AD [46,47]. By binding and stabilizing the structure of microtubule, tau regulates the assembly, spacing, and axonal transport of microtubules [48,49]. Through this, tau promotes the growth of nerve axons and helps the iron removal function of APP in nerve cells [50,51]. On the other hand, when excessive phosphorylation of tau occurs, the binding force between tau protein and microtubule is weakened, thereby weakening the structure of the neuronal cytoskeleton [52]. In addition, hyperphosphorylation of tau by GSK-3β (glycogen synthase kinase-3β) induces LTD (long-term depression) and slower membrane potential oscillations [53,54].

There was no direct effect on the survival or the development of neural tissue in tau protein-lacking mice [55]. However, tau has an important function in maintaining brain function, as changes in neuroplasticity occur by accumulation of iron in the brain and deterioration of LTD in tau knockout mice [50,56,57]. Malfunctions related with neuronal excitability, oxidative stress protection, and insulin resistance regulation were also reported [58,59,60]. However, the general view is that increasing or modifying tau protein converts tau into a toxic substance [61]. When hyperphosphorylation occurs in tau protein, the microtubule-binding domains bind to each other and become entangled [62]. This condition is called tauopathy, and it is a common pathologic finding in diseases with cognitive dysfunction such as Down’s syndrome, familial Alzheimer’s, fronto-temporal dementia, and senile dementia [61,63].

### 3.2. Pathological Processes in the Formation of Senile Plaques and Neurofibrillary Tangles

**Figure 2:**
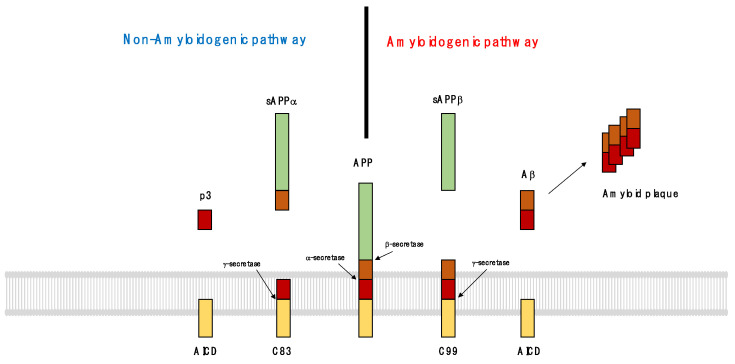
Non-Amyloidogenic and Amyloidogenic pathways of APP. In the non-amyloidogenic pathway, α-secretase cleavage of the Aβ region produces sAPPα and C83 fragments, and then γ-secretase cleavage of C83 produces p3 and AICD fragments which precludes Aβ formation. In the amyloidogenic pathway, cleavage of the Aβ region produces sAPPβ and C99 fragments. Then, the C99 fragment is cleaved by the γ-secretase complex into Aβ and AICD fragments. Accumulation of Aβs in the tissue forms plaque.

AD was diagnosed by observing the pathological findings of senile plaques and neurofibrillary tangles in postmortem brain tissue [64]. Senile plaques are formed by Aβ fragments fibrilized in the extracellular matrix around cells (Figure 2). Aβ is an APP fragment cut by β and γ-secretase. When APP is cleaved by α-secretase, it is divided into sAPPα and a membrane-tethered β-terminal fragment (C83), and then γ-secretase cleaves the C83, which is then divided into p3 and AICD (amyloid protein intracellular domain). This process is called ‘non-amyloidogenic pathway’ because processed fragments are easily removed and do not accumulate in the brain. However, by β-secretase, sAPPβ and α-C-terminal fragment (C99) are produced, and by γ-secretase, C99 is divided into Aβ and AICD. Aggregated Aβs accumulate in the brain by forming amyloid plaques; this process is known as the ‘amyloidogenic pathway’.

**Figure 3:**
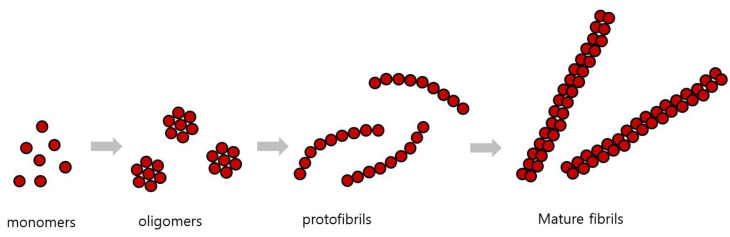
Aggregation of Aβ monomers to higher order oligomers, protofibrils and mature fibrils. Aβ monomers can form assemblies ranging from low molecular weight oligomers (dimers to pentamers) to mid-range molecular weight oligomers (hexamers to dodecamers). These oligomers can further aggregate into protofibrils and fibrils.

According to the cleavage site of γ-secretase, Aβ is made into fragments of several sizes, which are then processed into sizes of 40 and 42 amino acids. These Aβ monomers combine to form oligomers, protofibrils, and amyloid fibrils of various sizes (Figure 3). Amyloid monomers and oligomers are soluble in water and can spread out from the brain, but when large amyloid fibril is formed, it does not dissolve well and is deposited. These amyloid plaques are the main component of senile plaques found in the brains of AD patients [65]. That these aggregates of Aβ cause neurotoxicity and dementia is the ‘amyloid cascade’ hypothesis [66].

Aβ monomer aggregates in two ways; non-metal-dependent and metal-catalyzed. Aβ conjugates made without metal catalysis can be melted and decomposed, but metal catalyzed Aβ conjugates are difficult to decompose because they contain ionic and covalent bonds between Aβ fragments. These Aβ aggregates can catalyze the binding of other Aβ fragments as a aggregating core [67].

Aβ aggregation can get accelerated by other amyloidogenic proteins [68]. In particular, IAPP (islet amyloid polypeptide) is a peptide hormone secreted from pancreatic β-cells that aid the action of insulin. In 90% of type 2 diabetes patients, plaques containing IAPP as the main component are observed in the pancreas [69]. According to the ‘seeding-nucleation model’ of plaque formation, a nucleation seed is formed first, and as Aβ continues to attach to the seed, and the plaque is formed and grows [70]. When IAPP increases in type 2 diabetes patients, IAPP can form a seed in the brain and provide as a nucleation core of Aβ plaque formation [71]. In addition, IAPP aggregate is observed in the brains of patients with both T2DM and AD [72]. This may explain why AD and diabetes often coexist.

As fibrils and senile plaque builds up, Aβ binding also affects the kinase/phosphatase activity of tau, leading to hyperphosphorylation of tau and formation of neurofibrillary tangles (NFTs), and consequently to the dysfunction of neuronal synapses, and dementia [73,74]. When Aβ binds to the neuronal cell membrane, lipid peroxidation occurs and the toxic aldehyde 4-hydroxynonenal is produced. This leads to ATPase, glucose transporters, and glutamate transporters dysfunction, depolarization of cell membranes, excessive intracellular calcium influx, and mitochondrial damage [75]. Senile plaques and neurofibrillary tangles are mainly produced in brain regions related with memory, learning, and emotional behavior, such as the hippocampus, amygdala, entorhinal cortex, and basal forebrain [76]. In addition, Aβ itself increases reactive oxygen species, induces chronic inflammatory state by stimulating microglia, and activates mitochondria fission protein to induce mitochondria fragmentation. Additionally, mitochondrial Aβ is a proapoptotic factor which interacts with Aβ-binding alcohol dehydrogenase and cyclophilin D to induce neuronal cell death [77,78,79,80].

Amyloid plaques are formed outside the cells, but the fact that cytoskeletal proteins are mixed in as components of the plaque suggests that amyloid plaques are initially created inside the cells and then exit [81]. This is also related to the fact that β-secretase is mainly distributed in the cytoplasm. Somehow there must be a specific role of intracellular Aβ formation. In this regard, it is hypothesized that intracellular Aβ is a sort of inflammatory response to eliminate sources of infection [82]. Additionally, in a low metabolic state due to thiamine deficiency, BACE1 activity increases and results in more Aβ production [83,84].

There are several ways to prevent the accumulation of Aβ in the brain, including proteolytic degradation, cell-mediated clearance (which may itself involve proteolytic degradation), active transport out of the brain, and deposition into insoluble aggregates [85]. In particular, the activity of neprilysin, endothelin-converting enzymes, insulin-degrading enzyme (IDE), plasmin, and other Aβ-degrading enzymes are correlated with the accumulation of Aβ in the brain [86]. Neprilysin is the most effective Aβ degrading enzyme, acting mainly in the intraluminal/extracellular space, and it is also distributed in other cytoplasmic organelles such as the early Golgi and endoplasmic reticulum [87]. Endothelin converting enzymes 1 and 2 are distributed in the cell membrane and are responsible for removing Aβ out of the cell [88]. IDE degrades various peptide hormones such as insulin, glucagon, and amylin. It also degrades the intracellular domain of APP and Aβ, and it is estimated that it will play the most important role in the degradation of extracellular Aβ released from the cell [89]. Plasmin is a factor that activates the fibrinolytic cascade and degrades not only monomer but also fibril form of Aβ [90]. In addition, matrix metalloproteases (MMPs) such as MMP2 and MMP9, angiotensin-converting enzyme, cathepsin D, and an aspartyl protease have been found to have Aβ-degrading activity [91].

**Figure 4:**
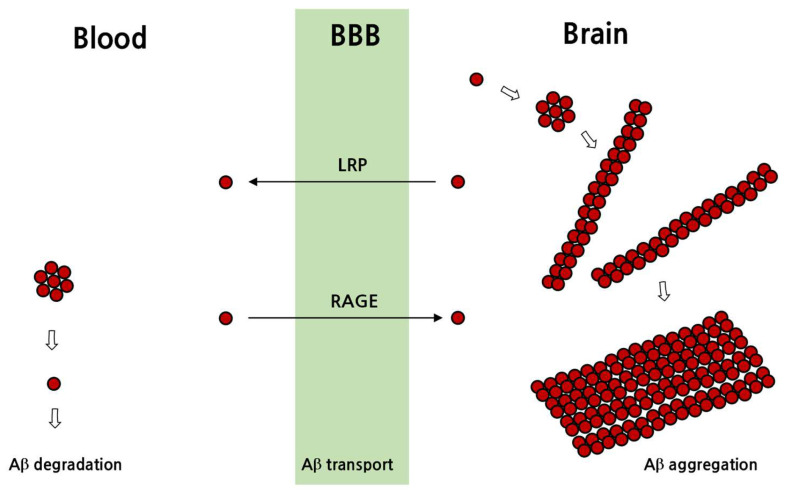
BBB transport of Aβ. Aβ is produced in the CNS, where it can aggregate into insoluble aggregates. Soluble Aβ can be transported across the BBB from brain to blook vis LRP. Aβ is also produced in peripheral tissues and can be transported from blood to brain via RAGE. LRP: low density lipoprotein receptor-related protein 1; RAGE: advanced glycation end product receptor.

In addition to the degradation of Aβ, extracellularly secreted Aβ is transported away from the brain to other tissues via blood, and chaperones such as apoE are involved in the transport of Aβ [92]. Aβ passage through the BBB is mediated by transporters such as advanced glycation end products (RAGE) receptor and low-density lipoprotein receptor-related protein 1 (LRP1) receptor [93,94]. In addition, glycoprotein 330 (gp330/megalin), P-glycoprotein receptors and the Aβ-binding proteins α2-macroglobulin, apoE and apoJ affect Aβ passage through the BBB [91]. In particular, RAGE moves soluble Aβ from the blood toward the brain to increase the amount of Aβ in the brain tissue, whereas LRP-1 mediates the movement from the brain to the blood [93,94] (Figure 4).

In addition, ApoE protein is related to the movement, removal, and aggregation of Aβ, and there are three isoforms: apoE2, apoE3 and apoE4. ApoE3 is the most common ApoE isoform, apoE4 is known to triple the incidence of AD, and apoE2 is known to reduce the risk of AD [95]. ApoE is mainly secreted from astrocytes and microglia into interstitial fluid, and high-density lipoproteins (HDL) containing apoE bind to Aβ secreted from neurons. The bound apoE/Aβ complex binds to the endocytic LDL receptor and flows into the cell. On the other hand, when the apoE/Aβ complex binds to heparin sulfate proteoglycans, it accumulates in the extracellular matrix and causes amyloid to accumulate outside the cell [96]. It appears that ApoE4 increases the incidence of AD by reducing amyloid clearance, and in astrocytes with ApoE4, the expression of LRP1 is decreased and thus Aβ clearance is also decreased [97].

NFT is a tangled mass of tau caused by excessive phosphorylation, and no longer constitutes microtubule material [98]. Tau normally acts as a cement that bonds block to block when making actin chains. However, when the tau is hyperphosphorylated, it sticks together to form a tangle rather than bonding between actin blocks. As a result, tau lesion can be characterized from two opposing aspects: by loss of function, in which the normal function of tau is lost, and by gain of function, in which the tangled tau results in toxicity [99]. In terms of loss of function, the movement of substances within the cell is not performed well, and iron ions may accumulate inside the cell due to the dysfunction of microtubules as a result of tau aggregation. Hyperphosphorylated tau has been shown to be toxic by itself [61]. As evidence, neurofibrillary tangles appear in hereditary diseases such as dementia with cognitive decline, Down syndrome, and early-onset AD [63]. In addition, the hyperphosphorylated tau in the cell is secreted out and can move into other cells, thereby spreading the tau lesion in the brain [100].

### 3.3. AD Treatment Development Status

Currently, there are six reagents made of five molecules that have received FDA approval as drugs that can be used for AD. Donepezil (Aricept^®^), approved in 1996, rivastigmine (Exelon^®^) in 2000, and galantamine (Razadyne^®^) in 2001 are acetylcholinesterase inhibitors [101]. Memantine (Nemenda^®^) was approved in 2003 and is a non-competitive NMDA receptor blocker that inhibits the excitotoxicity of neurons by blocking the inflow of calcium ions [102]. Namzaric^®^ is a mixture of donepezil and memantine. Then, in 2021, Aducanumab (Aduhelm^®^), an Aβ-binding antibody was added, resulting in six approved reagents. Among them, acetylcholinesterase inhibitors and the NMDA blocker only had the effect of temporarily alleviating the symptoms but did not prevent the progression of AD [103]. This means that the therapeutic mechanism of these reagents, which is to increase the concentration of acetylcholine in the brain or to reduce excitotoxicity, may not be the direct cause of AD. Based on the amyloid hypothesis, various methods have been tried to reduce the amount of Aβ accumulation in the brain. For example, preclinical and clinical trials are ongoing with materials such as Aβ binding inhibitors, Aβ polymer removing or inactivating antibodies, Aβ degrading enzyme activators, β-sheet structure degraders, and Aβ migration passage blockers [5].

For lesions caused by tau phosphorylation, tau kinase inhibitors (lithium, tidegulsib, tamoxifen) were tried, but had no proven clinical effect. Methylene blue, which inhibits tau oligomer and fibril formation, proceeded to phase three clinical trials for AD patients in 2016 [104,105,106]). On the other hand, immunotherapy to remove tau has been shown to be effective in animal studies, but its efficacy has not yet been proven clinically [107,108].

As of 2019, there are 132 candidates in phase 1, 2, and 3 clinical studies, and among them 28 candidates are in phase 3 clinical trials. Mechanistically, amyloid protein removal, BACE inhibition, amyloid and tau binding prevention, inflammation inhibition, and neuroprotective reagents are being tested [5]. In China, Green Valley’s GV-971, which is expected to improve cognitive function through the effect on intestinal microflora, received partial approval in 2019, but global phase three clinical trial is currently discontinued [109]. However, no drugs with proven efficacy existed until 2021, when Aducanumab was approved by the FDA. Therefore, to develop an effective treatment for AD, a new direction must be sought according to the cause of AD.

## 4. Why Insulin Resistance Is Linked to AD

AD is so closely related to diabetes that it is known as a so-called ‘type 3 diabetes’. Decrease in brain glucose metabolism occurs more than 10 years before the onset of AD symptoms [12]. Therefore, abnormal energy metabolism is pointed out as the cause of dementia. According to the results of a Whitehall II cohort study in the UK (n = 5653), memory decreased 45% faster, rational judgment ability declined 29% faster, and global cognitive ability to evaluate multiple cognitive functions using ACE-R score decreased 24% more rapidly in patients with diabetes [110]. In middle-aged populations with type 2 diabetes, the duration of illness and the degree of glycemic dysregulation had a significant effect on the rate of cognitive decline [110].

### 4.1. Brain Energy Mobilization

Among the organs of the body, the brain consumes one of the largest proportions of energy. Although brain mass constitutes 2% of total body weight, its glucose consumption in the resting awake state accounts for 25% of total body glucose used [111]. A significant amount of this energy is used to maintain the nerve cell membrane potential which is important for signal conduction [112]. The brain uses glucose, lactate, ketone bodies, amino acids, and short chain fatty acids (SCFAs) as energy sources, but glucose is used preferentially, and the others are utilized as alternative energy sources in the absence of glucose [113].

The brain uses 6–7 mg/100 g of glucose per minute, which is equivalent to 120–130 g per day [114]. Because glucose is a polar substance, it cannot freely pass through the cell membrane but enters the cell through a transporter called glucose transporter (GLUT). In the brain, GLUT1 and GLUT3 are the main glucose transporters, and are not insulin dependent type transporters [115]. Insulin is a pancreatic hormone that lowers blood glucose by acting on GLUT4 to transport glucose into cells. Insulin resistance is a phenomenon in which this action of insulin is weakened and blood glucose concentration increases [116]. In the brain, GLUT4 is far less prevalent than GLUT1 and GLUT3, which means that the brain is not an organ that stores glucose in response to insulin. However, it is known that GLUT4 is involved in glucose influx into synaptic areas during high synaptic activity conditions [117]. Therefore, although insulin resistance does not reduce the overall influx of glucose into brain tissue, it may affect synaptic activity.

Lactate is the product of anaerobic glycolysis and is mainly produced in skeletal muscles that require a rapid supply of ATP [118]. Lactic acid produced in the muscles enters systemic circulation and goes to the liver, where it is reconverted into glucose for recycling. Circulating lactic acid can also enter brain cells through the MCT (monocarboxylate transporter) channel to be used as an energy source [119]. Lactic acid is also produced by astrocytes during glucose metabolism to provide ATP for the conversion of glutamate into glutamine [120]. Lactic acid produced in astrocytes goes to nerve cells and is converted into pyruvate. Astrocytes play an important role in creating emotionally salient memory by synthesizing glycogen using glucose and supplying it to neurons by making lactate [121].

Ketone bodies are the general name of acetone, acetoacetate, and β-hydroxybutyrate. When blood sugar decreases, such as in continuous fasting, the liver breaks down triglycerides and produces free fatty acid and ketone bodies to provide an alternative energy source [122]. However, due to their long chain structure, fatty acids hardly pass the BBB, whereas ketones have a simple structure and can pass through the BBB. These ketone bodies go to nerve cells and are converted into acetyl-CoA and utilized for energy production [123].

Amino acid is the main component of the structure and function of a living body rather than an energy source. However, in a low energy state, by removing the amine group, an amino acid can be converted to acetyl-CoA or other intermediate metabolite of the TCA cycle. This process is called gluconeogenesis [124].

SCFA is a substance produced by decomposition of dietary fiber in the digestive tract by enterobacteriaceae, and three main types have been identified: acetate, propionate, and butyrate [125]. They are absorbed by the intestinal epithelial cells and utilized as energy sources. Butyrate is mostly consumed by enterocytes (gastrointestinal epithelial cells), propionate can reach to the liver and is generally utilized for gluconeogenesis by hepatocytes, and acetate reaches the blood circulation. Of these, butyrate has many known functions in the brain, but because it hardly reaches the circulation, the amount that passes through the BBB is little [126]. However, the amount of butyrate in the blood may increase depending on the intake of dietary fiber or the characteristics of enterobacteriaceae [127]. Currently, research on the effects of SCFAs and gut-brain interaction is being actively conducted.

**Figure 5:**
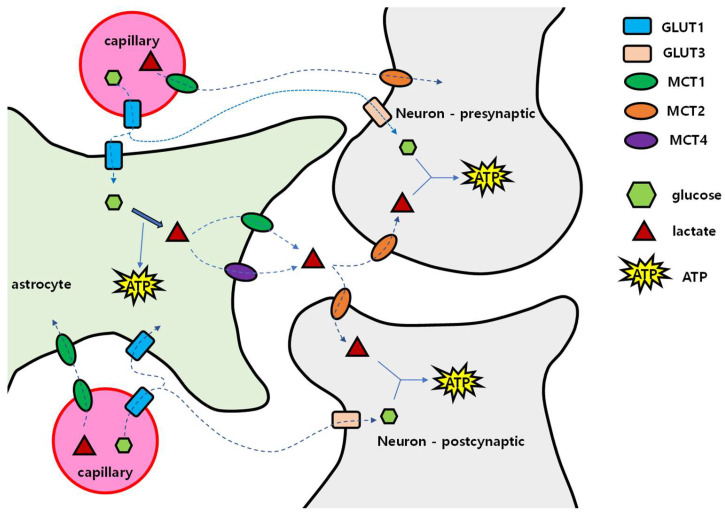
Glucose and lactate delivery to nerve cells. Glucose is transported from capillaries to astrocytes via GLUT1. After entering the astrocyte, glucose is metabolized to lactate, then lactate is transported out into the interstitium via MCT1 and MCT4. Lactate enters into presynaptic and postsynaptic neurons via MCT2 and is used for energy metabolism after being converted into pyruvate. Glucose can also enter neurons directly via GLUT3. Blood capillary lactate enters into astrocytes via MCT1 and into neurons via MCT2. GLUT: glucose transporters; MCT: monocarboxylate transporter.

For the delivery of these energy sources, passage is required through the neurovascular unit composed of capillaries and cells in the brain. This unit is composed of vascular endothelial cells, pericytes, basal lamina, astrocytes, and neurons [128]. The BBB, which consists of the tight junction of vascular endothelial cells, basement membrane, and end foot of astrocytes, can pass lipid-soluble substances, but water-soluble substances such as glucose, amino acids, and nucleic acids need transport proteins to transit through [129]. Once glucose has crossed the BBB, glucose can reach neurons through two pathways: via the extracellular fluid in brain tissue, and via astrocytes (Figure 5). In the extracellular pathway, glucose enters GLUT1 of the vascular endothelial cells, spreads into the extracellular fluid, and then enters neurons through GLUT3. In the astrocytic pathway, after passing through GLUT1 of vascular endothelial cells, glucose enters astrocytes through GLUT1, and is converted to glycogen for storage or to lactic acid by glycolysis. Then, lactic acid exits into extracellular fluid by MCT1 or MCT4. In the extracellular fluid, lactic acid enters the nerve cell through MCT2. This is called the ‘astrocyte–neuron lactate shuttle’.

Concentrations of glucose and lactic acid in the brain extracellular fluid are much lower than in blood, which means that concentration gradient may drive the diffusion of these molecules [130]. Then, if the concentration of glucose and lactic acid in the blood increases, the concentration of glucose and lactic acid in the cerebrospinal fluid will also increase. However, in patients with type 2 diabetes, the glucose concentration in the cerebrospinal fluid was lower than in normoglycemic persons, which may be because systemic insulin resistance induced intracerebral insulin resistance [131]. Additionally, in the glycolysis process of glucose, enzyme activity is regulated through several steps. Hexokinase initiates the glycolytic process by converting glucose into glucose-6-phosphate, and when this enzyme is saturated (Km = 0.05 mmol/L), glycolysis no longer occurs even when the amount of glucose increases. On the other hand, the reaction of lactate conversion into pyruvate only requires NAD^+^ and can be used for ATP synthesis [132]. Unlike glucose, lactic acid has no restriction on conversion to pyruvate, so it is useful as a continuous energy source for nerve cells.

Astrocytes convert glutamate into glutamine [133]. The energy required for this reaction comes from the metabolism of glucose to lactate. Therefore, when the glucose supply to astrocytes is reduced, excitotoxicity may occur due to poor glutamate removal from excitatory synapses. Memantine, an NMDA antagonist approved for AD treatment, may suppress excitotoxicity in a status of excessive synaptic glutamate which may be a consequence of insufficient glucose [134]. Generally, among the cells in the brain, only astrocytes synthesize and store glycogen, and energy production by gluconeogenesis which uses aspartate, glutamate, alanine, and lactate only occurs in astrocytes but not in neurons [135]. Nerve cells are vulnerable to a low energy supply and therefore depend on other cells in the brain tissue.

### 4.2. When and How the Brain Deals with the Lack of Glucose

In AD, hypometabolic changes precede the appearance of dementia symptoms by a decade or more. There are other conditions associated with brain hypometabolism such as Beriberi. Beriberi is a hypometabolic state due to the lack of thiamine (vitamin B1) [136]. Thiamine is a material used for the synthesis of thiamine diphosphate (TPP), and TPP is required for the enzymatic activity of pyruvate dehydrogenase complex and the ketoglutarate dehydrogenase complex. These two enzyme complexes play a key role in oxidative phosphorylation. The pyruvate dehydrogenase converts pyruvate to acetyl-CoA, and the ketoglutarate dehydrogenase converts α-ketoglutarate to succinyl-CoA; these two CoAs are the intermediate molecules of the TCA cycle. Therefore, when thiamine is insufficient, these enzymes do not work, and the cellular energy factory producing ATPs shuts down. Another enzyme that uses TPP as a coenzyme is transketolase, which mediates the non-oxidative pentose phosphate pathway (PPP) [137]. The PPP makes hexose, a constituent of DNA and RNA, from glucose. That is to say, thiamine deficiency causes a shortage of materials for DNA and RNA synthesis.

The causative factors for Beriberi include long-term malnutrition due to alcoholism, malabsorption of thiamine, pregnancy, increased thiamine requirement due to hyperthyroidism, decreased thiamine absorption due to liver disease, and loss of thiamine due to dialysis or chronic diarrhea [138]. Thiamine deficiency causes memory impairment, amyloidosis and hyperphosphorylation of tau in the brain [84,139]. Severe Beriberi, called Wernicke–Korsakoff syndrome, results in permanent damage of the central nervous system, and mainly occurs in chronic alcohol abuse patients [140]. In animal studies, thiamine deficiency induced dementia-like conditions such as decreased neurogenesis, memory loss, and plaque/tangle formation [141]. In AD patients, the amount of thiamine was lowered by one-third compared to age matched normal controls [142]. Additionally, the concentration of thiamine gradually decreases with age [143]. The cause of the decrease in thiamine concentration with age is not yet clear, but it was suggested that a gradual decrease in intestinal alkaline phosphatase activity may lead to diminished thiamine absorption [144].

When the amount of glucose in the bloodstream decreases, the liver breaks down stored fat to make ketone bodies, which become the main energy source for the brain [145]. However, if the supply of ketones is inadequate, brain cells break down the myelin sheath, which contains a lot of lipids, to produce ketones on their own. This process may lead to the decrease in white matter volume that occurs with age [146]. Additionally, the decrease in white matter volume becomes more pronounced as dementia progresses, suggesting that dementia is associated with a lack of energy in the brain [147].

Changes in energy metabolism in the brain also occur during the normal aging process. In the brain, ATP is synthesized in astrocytes and neurons, with astrocytes primarily using the glycolysis pathway and neurons producing ATP through oxidative phosphorylation [148]. However, the brain under cellular stress requires more energy, and to supplement the insufficient ATP, the astrocytes increase glycolysis to produce and secrete more lactate into the brain interstitium [149]. Neurons absorb and convert this lactate into acetyl-CoA to make ATP through oxidative phosphorylation. Among these neurons, some maintain a normal state, but others with oxidative damage have a compensatory increase in oxidative phosphorylation, and were described by Demetrius et al., as type 2 neurons [150]. When the supply of lactate is increased, these type 2 neurons synthesize more ATP, which is called the ‘inverse-Warberg effect’ and appears in aged neurons [151]. However, due to mitochondrial damage, more oxygen free radicals are made and more oxidative damage to the surrounding cells occurs [150]. When such damage accumulates, neuronal functions deteriorate as evidenced by decreased ATP production and the pathological process progresses as the function of the cells gradually decreases. Sporadic AD is also suggested as a disease related to insufficient energy metabolism [152].

Cortical hypometabolism also appears before the onset of AD in the ApoE4 carrier, which has been pointed out as a genetic factor associated with the onset of AD [153]. Among the three types of human apolipoprotein Es, ApoE4 showed the lowest intracerebral glucose uptake and metabolism [154]. Additionally, the upregulation of oxidative phosphorylation in neurons happens more in the ApoE4 carriers [155]. On the other hand, intracerebral movement of ketone bodies was the least with ApoE3. This may be related to the reason why ApoE2 has the lowest incidence of AD, and ApoE4 carriers have increased AD incidence [156].

Enolase, an enzyme whose activity was changed in MCI and AD, is involved in glucose metabolism [157]. Enolase converts 2-phosphoglycerate to phosphoenolpyruvate in the glycolysis pathway. Interestingly, enolase activity was increased in MCI, early onset AD, and AD [158,159]. The increase in enolase activity may be an adaptive process in which glucose is more efficiently decomposed and supplied as an energy source in a state of insufficient glucose. In addition, enolase increases tPA binding and increases plasmin, and this can degrade Aβ [160]. Therefore, the increase in enolase activity may be a sign of abnormal brain metabolism and can be seen as an effort to eliminate Aβ.

## 5. Insulin and Insulin Resistance

Insulin resistance refers to a condition in which insulin is produced in the pancreas, but the bioactivity in the target organ of insulin is reduced. When insulin resistance increases, the glycemic control function of insulin decreases, and the intracellular insulin signaling process deteriorates [161].

### 5.1. Synthesis, Actions, and Degradation of Insulin

Insulin is a peptide hormone made in pancreatic β cells composed with 51 amino acids [162]. Increase in blood glucose, amino acids, acetylcholine, cholecystokinin, and incretin hormones stimulate synthesis and secretion of insulin [163,164]. The well-known action of insulin is glycemic control exerted when insulin mediates the entry of glucose into skeletal muscle cells and adipocytes through GLUT4 and inhibits glucose production in hepatocytes by blocking glycogenolysis in hepatocytes, thereby reducing the amount of glucose in blood [162]. In addition, as an anabolic hormone, insulin promotes the intracellular absorption of amino acids and fatty acids and inhibits catabolic process such as gluconeogenesis, glycolysis, lipolysis, and proteolysis [165].

**Figure 6:**
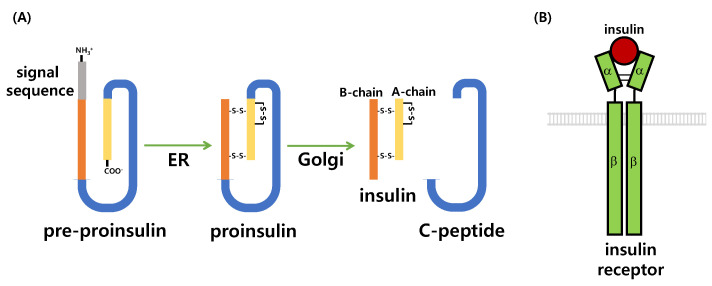
Insulin synthesis and insulin receptor. (**A**) Insulin biosynthesis begins as a precursor, preproinsulin, within the pancreatic β cell cytosol. Preproinsulin is comprised N-terminal signal sequence (gray), B-chain (orange), C-peptide (blue), and A-chain (yellow). Preproinsulin translocates into the endoplasmic reticulum and by cleavage of the signal sequence, forms proinsulin. The proinsulin folds by forming 3 disulfide bonds then trafficking through Golgi complex into secretory granules. Prohormone convertase and carboxypeptidase E processes proinsulin to C-peptide and mature insulin composed with A- and B-chain. ER: endoplasmic reticulum. (**B**) Insulin receptor is a dimer of identical units that span the cell membrane. Each of the 2 subunits is made of α-chain and β-chain, connected by a single disulfide bond. The α-chain is located extracellular and binds with insulin. The β-chain spans the cell membrane and has a tyrosine kinase domain which is activated when insulin binds to the α-chain.

The initial form of insulin, preproinsulin, gains insulinic activity after the cleavage of signal peptide and c-peptide (Figure 6). Insulin binds to the α subunit of the insulin receptor located at the cell membrane, and by the insulin binding, two insulin receptors approach each other in proximity, then the β subunits phosphorylate each other, which in turn activates the insulin receptor substrate (IRS) adapter protein, PI3K, and AKT to transmit intracellular signals [166]. There are two types of IRS: IRS1 and IRS2 [167]. IRS1 is common in skeletal muscle, adipose tissue, and cerebral cortex; IRS2 is abundant in liver and hypothalamus. Activation of IRS exhibits various cellular actions such as sending GLUT4 to the cell membrane to allow more entry of glucose into the cell. However, it also triggers intracellular signaling process by activating signaling molecules such as mTOR, GSK-3β, CREB, filamin A, and NOS (nitric oxide synthase) [168]. In addition, SHC (src homology and collagen) protein is phosphorylated by the β subunit of the insulin receptor and continues the activation of RAS-RAF-MAPK pathway, thereby affecting the transcription, translation, and post-transcriptional modification of transcripts [169]. As such, insulin exhibits very diverse effects, and these effects are specific to tissues and cell types.

IDE is the insulin degrading enzyme and its activity increases by an excess of insulin [170]. IDE can decompose Aβ, so if the IDE activity is sufficient, it will breakdown and aid in the removal of Aβ out from the brain, whereas in a lack of insulin state, Aβ can accumulate [171].

### 5.2. How Does Insulin Resistance Emerge?

When blood glucose increases, pancreas secretes insulin to (1) suppress gluconeogenesis in the liver, (2) increase glucose absorption in muscles and adipose tissue, and (3) suppress lipolysis in adipose tissue [161]. Through these insulin-mediated effects, glucose released from the liver into bloodstream reduces, and glucose uptake into muscles and adipose cells increases, resulting in the reduction in blood glucose concentration. When insulin resistance develops, liver, muscle and adipose cells do not respond to insulin, and blood glucose concentration increases. Then, the pancreatic β cells try to compensate by secreting more insulin into the bloodstream, resulting in hyperinsulinemia. However, type 2 diabetes is a condition in which blood glucose levels do not fall despite hyperinsulinemia [172].

Disruption at any of the insulin signaling event weakens the action of insulin, such as insulin receptor α-subunit binding, phosphorylation of β-subunit, IRS, PI3K, PKD1, and AKT [173]. Intracellular biochemical factors associated with insulin resistance are (1) IRS-1 dephosphorylation due to increased PTPIB (protein-tyrosine phosphatase-1B) activity [174], (2) increase of inflammatory mediators such as TNF-α, MCP-1, CRP, interleukin, (3) activation of IKKβ/NF-κB and JNK pathways due to oxidative stress and consequent degradation of IRS [175], (4) decreased IRS-1 activity due to the phosphorylation of serine 307 [176], (5) mitochondrial dysfunction [177], (6) decreased number of insulin receptors or GLUT4 mutation, (7) ER stress and etc. [178].

Long-term intake of a high-calorie diet weakens this intracellular insulin signaling process [179]. This is related to the increase in the synthesis of triglycerides, the accumulation of fat, and the decrease in the expression of insulin receptors and intermediates of the insulin signaling pathway. A high-fat diet especially induces insulin resistance by altering the composition of membrane lipids, particularly ceramide [180]. Long-term intake of a high-fructose diet facilitates the accumulation of visceral fat, and a long-term high-protein diet stimulates pancreatic insulin and glucagon secretion [181,182]. In the obese state, the expression and activity of protein tyrosine phosphatase (PTPs) are increased, thereby reducing the activity of intermediate molecules in the insulin signaling process [183].

Substances that cause insulin resistance in cells are attributed to the accumulation of long-chain acyl CoAs such as lysophosphatidic acid, phosphatidic acid, diacylglycerol (DAG), sphingolipids such as ceramide and GM3, and phospholipids such as lysophosphatidylcholine rather than triglyceride [184]. Increased DAG in muscle and liver activates PKCs (muscle type: PKCθ, liver type: PKCε), JNK, and IKK-β. As a result, activated serine/threonine kinases increase serine phosphorylation of IRS1 & 2 and decrease tyrosine phosphorylation. As a result, the activity of PI3K and Akt is reduced, resulting in decreased glucose inflow by GLUT4 in the muscle, and hepatic glucose output is not suppressed in the liver, resulting in increased blood sugar [185]. In addition, ceramide, a lipid component constituting the cell membrane and usually synthesized from palmitic acid, is produced by TNFα activated sphingomyelinase [186]. Ceramide activates protein phosphatase 2 (PP2) to dephosphorylate Akt and activates JNK1 and IKKβ and induces serine phosphorylation of IRS1 & 2 which weakens the intracellular insulin signaling [187].

Stress hormones and inflammatory cytokines also cause insulin resistance [188]. Inflammatory cytokine and ER stress phosphorylates serine of IRS-1 (serine 302 and serine 307), which inhibits IRS activity and results in insulin resistance [189]. Activated IKK-β promotes the expression of genes that induce insulin resistance. TNF-α, IL-6, IL-1β, and resistin induce IRS1,2 degradation, and in the liver, dephosphorylate insulin receptor and IRS through STAT3. These paths are how fatty acids, inflammation and glucocorticoids affect the development of insulin resistance [190].

### 5.3. Insulin Action on the Brain

Insulin receptors are distributed in many cell types such as hepatocytes, skeletal muscles, and adipose cells which are glucose depots by uptaking glucose from the blood [191]. However, the brain is not a glucose reservoir despite its rich distribution of insulin receptors. Instead, insulin receptors trigger intracellular signaling which are important in maintaining brain functions [192]. Insulin contributes to promoting neuronal neurite outgrowth, regulating release and reuptake of catecholamines, and regulating ligand-gated ion channel trafficking, GABA, NMDA, AMPA receptor membrane trafficking, LTP and LTD induction, dendritic spine formation, apoptosis suppression, and neuronal survival promotion [193]. Through these roles, insulin promotes learning and memory, synaptic density, and connectivity in neurons as a growth factor [194].

Administration of insulin has been shown to enhance performance in a passive-avoidance memory task and spatial memory training altered hippocampal expression of insulin receptors [195,196]. Intranasal insulin enhances memory in the healthy, MCI, and AD patients [197]. Systemic administration of insulin increased the plasma concentration of Aβ_1-42_ by promoting their brain-to-blood transport through the BBB. This was related with an increased expression of LRP1 by insulin, which plays an important role in Aβ clearance [198].

Insulin also affects energy metabolism in the brain. Neurons receive glucose mainly through GLUT3, which is opened by depolarization of NMDA receptors [199]. As insulin receptors are found both in presynaptic and postsynaptic regions, insulin may affect the uptake of glucose into neurons [192]. It was shown that reduced plasma insulin precedes decreased GLUT3 translocation in the hippocampus of aged AD mice [200]. However, since GLUT4 is also distributed in the synapse, insulin increased glucose inflow when energy demand increased due to high synaptic activity [7,201]. GLUT4 was also found in the hypothalamus, and in mice with GLUT4 knockout, the ability to monitor the amount of glucose deteriorated resulting in poor blood sugar control [201,202].

Appetite control by hypothalamus is also affected by insulin. When the hypothalamic IRS2 is activated by insulin, appetite, glucose synthesis in the liver, lipolysis in adipocytes, BCAA catabolism in the liver, and fatty acid release from liver to blood all decrease. Consequently, blood sugar decreases, which is independent on the peripheral actions of insulin [203,204]. Therefore, a decrease in brain insulin leads to an increase in appetite, glucose synthesis and lipolysis [205]. Insulin also increases the connectivity between the DMN (default mode network) and task area [206]. However, insulin resistance reduces connectivity between these brain circuits, a phenomenon suggested as one of the causes of depression.

At the cellular level, insulin signaling affects vascular endothelial cells, neurons, glial cells and pericytes. Insulin acts on vascular endothelial cells to regulate capillary constriction and relaxation [207]. A low concentration of insulin causes vasoconstriction, and a high concentration of insulin relaxes the blood vessels [208]. Through this, the BBB structure and discharge of Aβ from the brain tissue into the blood vessels is maintained. Thus, insulin resistance causes impaired cerebral blood pressure regulation, increased BBB permeability, and increased intracerebral Aβ [209]. In neurons, insulin regulates learning, memory, and synaptic plasticity through the regulation of synaptic signaling [210]. Therefore, by insulin resistance in the hippocampus, impairment of long-term memory occurs. Additionally, as insulin inhibits tau phosphorylation, and activated IDE can degrade Aβ, lack of insulin inhibits the formation of long-term memory, induces tau-hyperphosphorylation, and increases intracranial Aβ [211]. In astrocytes, insulin regulates glycogen synthesis, astrocyte proliferation, maintenance of mitochondria function, expression of ApoE and GLUT1, and passage of glucose through the BBB [212]. Therefore, insulin resistance decreases glycogen storage, inhibits astrocyte proliferation, decreases glucose influx into the brain due to reduced GLUT1 expression, and increases ApoE expression. In an animal model of insulin deficiency, the number of astrocytes decreases, BBB is broken, expression of GLUT1 is reduced, and the movement of glucose to the brain is reduced, so that the main fuel of astrocytes is shifted from glucose to lipids [213]. Insulin stimulates pericyte proliferation which inhibits the death of vascular endothelial cells and increases insulin sensitivity of neurons [214]. Insulin resistance decreases the number of pericytes, decreases the number of vascular endothelial cells, and increases insulin resistance in neurons. Insulin-induced Akt signaling is also involved in the regulation of oligodendrocyte proliferation, survival, differentiation, and myelination, and is involved in the inflammatory response of microglia [215,216].

### 5.4. Brain Insulin Resistance

Insulin resistance in the brain is a condition in which (1) the amount of insulin in the brain decreases or (2) brain cells do not respond to insulin [193]. Because the brain does not act as a storehouse of glucose in hyperglycemic conditions, brain insulin resistance does not necessarily lead to a decrease in glucose concentration in the brain. Instead, changes in cellular function or synaptic activity occur due to weakening of the insulin signaling process, such as a decrease in the influx of insulin or a decreased numbers of insulin receptors [217].

Insulin found in brain flows from plasma through the BBB by transporters, and the amount of the influx is proportional to the plasma insulin concentration [218]. There is evidence that brain cells can produce insulin, but the phenomenon has not yet been definitively confirmed [219,220]. The BBB permeability of insulin is reduced by obesity, inflammation, hyperglycemia, diabetes, and dyslipidemia [221,222]. The ratio of insulin concentration in cerebrospinal fluid to plasma decreases with age, when there is systemic insulin resistance, and in AD patients [223,224,225]. In contrast, when insulin sensitivity is restored by weight loss the amount of insulin in CSF is increased [226]. Insulin receptor numbers also decrease with age and by insulin resistance [173]. A reduced number of insulin receptors in vascular endothelial cells results in less influx of insulin into the brain and weakened nerve activity [223]. In particular, cognitive function gradually deteriorates in type 2 diabetes, while intranasal insulin administration improves cognitive function in diabetes patients [227,228]. A decrease in muscle mass is an important cause of the gradual increase in insulin resistance associated with age. This is because skeletal muscles are the main glucose absorbers in response to insulin, and when muscle mass decreases, blood glucose drops less, which leads to increased insulin resistance [229].

Molecular factors that are related to insulin resistance in brain are increased activity of PTP1B, proinflammatory cytokines, reactive oxygen species, GLUT4 dysfunction, etc. [230]. PTP1B inhibits insulin signaling by dephosphorylating IRS-1 and inhibits its activity [231]. In the presence of insulin resistance, proinflammatory cytokines such as TNF-α, monocyte chemotactic protein-1 (MCP-1), C-reactive protein (CRP), and interleukins are increased. TNF-α inhibits IRS-1 activity and suppresses GLUT4 expression [232,233]. IL-1 reduces IRS-1 expression, and IL-6 stimulates IRS degradation [234]. NO from activated iNOS suppresses PI3K-AkT signaling [175]. Oxidative stress associated with accumulation of ceramide and protein aggregates, activation of pro-inflammatory cytokines, mitochondrial dysfunction, as well as neuronal apoptosis is linked to brain insulin resistance [235]. Free radicals induce degradation of IRS through NF-κB and JNK pathways and inhibit GLUT4 trafficking to the cell membrane [236,237]. Studies have also shown that oxidative stress enhances the accumulation of Aβ-peptides and diminishes dendritic spine density and long-term potentiation (LTP) [235].

## 6. How Insulin Resistance Causes Dementia

Due to insulin resistance, AD-related pathological process progresses due to (1) the amount of glucose is insufficient in the brain and (2) the intracellular insulin signaling is weakened.

### 6.1. Brain Glucose Insufficiency

The storage capacity of glucose in the brain is limited, so when the glucose supply is reduced, brain function deteriorates rapidly [238]. Vanitallie (2013) showed that hypometabolic state of brain glucose precedes cognitive symptoms in dementia [239]. Glucose consumption reduces first in the hippocampus, followed by the posterior cingulate cortex, parietal lobe, and frontal lobe [240]. Additionally, there is an apparent decrease in glucose metabolism in hippocampal structures based on FDG-PET scan images in MCI patients [241]. In normal aged individuals, sometimes glucose metabolism is decreased but not always, which leads to the speculation that the decreased glucose metabolism in MCI is likely pathologic rather than an aging process [238]. Furthermore, it was found that the decrease in glucose metabolism in AD patients was proportional to the decrease in GLUT1 expression [242]. As a mechanism for this, insulin resistance due to high-glycemic diet decreased brain GLUT1 expression and induced AD-like symptoms [243,244]. Glucose intolerance resulting from inactivation of both insulin receptor and IGF-1 receptors in the hippocampus or central amygdala caused cognitive deficits and anxiety [245].

When energy metabolism in the brain decreases, BACE1 activity increases and results in more Aβ accumulates [246]. Similar lack of energy metabolism is seen in thiamine deficiency, which causes an increase in BACE1 activity and Aβ accumulation [141]. Decreased activity in the electron transport chain due to the lack of intracerebral glucose also leads to increased production of APP and accumulation of Aβ [247]. ROS overproduction due to mitochondrial dysfunction enhances the accumulation of Aβ and induces oxidative damage to proteins, lipids, and nucleic acids [1,247].

Impaired glucose metabolism is also associated with excitotoxicity as a result of reduced glutamate uptake in astrocytes [248,249]. As NMDA receptor antagonist memantine has been shown to improve AD patients’ cognitive functions, excitotoxicity may have a role in AD pathology [250]. It was also shown that Aβ oligomers interact with NMDA receptor to exert neurotoxicity [251]. In post-mortem AD brains, amyloid plaques, NFTs are co-localized with excitatory pyramidal neurons which support the notion that excitotoxicity is involved in the pathological mechanism of AD [252].

When the amount of intracellular glucose decreases by reduced GLUT1 and GLUT3 expression, the amount of uridine 5’-diphosphate-N-acetylglucosamine (UDP-GlcNAc), which is a product of glucose metabolism, is reduced [253]. UDP-GlcNAc binds to tau and APP by O-GlcNAc transferase (OGT), and O-GlcNAcylation prevents phosphorylation of these proteins. Therefore, when the amount of UDP-GlcNAc is decreased, hyperphosphorylation of tau and APP can occur, resulting in neurotoxicity [254]. Because the reciprocal changes in O-GlcNAylation and hyperphsohorylation of tau responds rapidly to glucose availability, decreasing brain glucose uptake could contribute to the development of AD [255].

Decreased white matter density is another early sign of AD and MCI [256]. Changes in the intensity of white matter are also related to energy metabolism [257]. The areas where these changes occur mainly are the cingulum bundle, uncinate fasciculus, and superior longitudinal fasciculus of MCI patients, which are the areas corresponding to the DMN [258]. In particular, the cingulum bundle connects the hippocampal formation, prefrontal cortex, and posterior cingulate cortex [259]. Patients with MCI also have hypometabolism in the PFC and posterior cingulate cortex, which are also the main components of DMN [258]. Connectivity between these areas is provided by the superior longitudinal fasciculus [257]. Therefore, degeneration of the cingulum and hypometabolism of the prefrontal cortex are associated with hippocampus atrophy and cognitive decline [260,261]. When glucose is insufficient, ketone bodies are used as an alternative brain energy source [262]. This loss in white matter integrity could be a direct result of a switch from the use of ketone body supplied from the peripheral ketogenic organ, the liver, to ketone body resulting from local myelin breakdown via FA oxidation by astroglia [122]. In the prodromal AD brain, ketogenic enzyme such as SCOT (3-ketoacid CoA transferase) is upregulated [263]. Since insulin suppresses ketone body production in the liver, in a systemic insulin resistant state, ketone supplement to the brain is not enough. Then, the brain cells try to obtain an energy source through catabolic gluconeogenesis [264]. Decreased mitochondrial efficiency, increased oxidative stress and H_2_O_2_ overproduction activate PLA2 (phospholipase 2) and result in myelin sheath degradation for use of fatty acid and ketogenesis [122]. If this process continues for a long time, the myelin sheath of the white matter decreases, and the speed of nerve transmission through action potentials between neurons also decreases [265]. Free radical induced mitochondrial damage and accumulation of Aβ accelerate this process of energy anomaly as a vicious cycle [266]. Alternately, lesions in white matter integrity may be caused by inadequate lipid synthesis due to competition between consumption of ketones/acetyl-CoA for bioenergetics and lipid synthesis [122].

Impaired glucose metabolism could induce inflammatory responses and exacerbate AD pathology. Pathophysiological cascades of inflammatory responses are associated with mitochondrial dysfunction and oxidative stress, excess of inflammatory factors, excitotoxicity, AGEs, apoptosis, hyper-activation of protein kinases, etc. [84,267,268,269,270,271]. Additionally, glucose metabolism is necessary for autophagy. Autophagy is responsible for the degradation of folded proteins in cells, and its dysfunction could lead to Aβ aggregation and tauopathy [272]. The initiator of autophagy process, Beclin 1, was decreased in AD patients [273]. The mammalian target of rapamycin (mTOR) pathway receives autophagic stimuli and signals to initiate autophagy [248]. Enhanced mTOR signaling activity increased Aβ deposits and NFT formation while inhibition of mTOR by rapamycin reduced Aβ pathology by increasing autophagy [274,275]. Impairment of autophagy also increased β- and γ-secretase activities and contributes to the tauopathy [248,276].

### 6.2. Brain Insulin Insufficiency

As the blood insulin concentration increases, the brain and CSF insulin concentrations also increase correspondingly, but in a prolonged hyperinsulinemia state, insulin receptor of the BBB downregulates and insulin transport into the brain reduces [277]. Insulin receptors are distributed in astrocytes and neuronal synapses, especially in the olfactory bulb, cingulate cortex, hippocampus, hypothalamus, amygdala and septum [278].

Based on the results from the previous studies, insulin receptors, insulin-like growth factor receptors, and IRS-1 participates in the pathogenesis of AD [279]. Steen et al., (2005) has demonstrated that the expression of insulin and IGF-1/2 receptors were markedly reduced in AD brains in correlation with the pathological alterations of AD such as increased GSK-3β activity and APP mRNA expression [280]. Moreover, IRS-1 was suppressed in the hippocampus, and the degree of suppression was proportional to the amount of senile plaque and cognitive decline [281]. Accordingly, intranasal insulin injection improved cognitive function in AD patients [282].

As the brain is an insulin responding organ, insulin resistance correlates to cognitive dysfunction [283]. When the brain has a decreased number of insulin receptors, hyperphagia, insulin resistance, central hypogonadism, impaired response to hypoglycemia, and depression-like behaviors appear whereas loss of IGF-1 receptor caused impaired brain development [284,285,286,287]. Insulin resistance also results in the lack of trophic support. In the brain, insulin/p-IRS/PI3K/Akt signaling enhances neurite outgrowth and synaptogenesis via upregulating BDNF and PSD-95 [288,289]. BDNF promotes synaptic plasticity through CaMKII, synaptophysin and PSD-95 [290]. In an insulin resistant condition, activation of microglia and astrocytes continues, and these cells secrete glia-derived proinflammatory cytokine which lowers BDNF levels [291,292,293]. Insulin resistance model animals showed impaired hippocampal neuroplasticity which is characterized by an increase in PI3K p85 subunit autophosphorylation with a decrease in phospho-Akt [194,294]. When insulin receptors and IGF-1 receptors were inactivated in the hippocampus (Hippo-DKO), GluR1 expression decreased and increased anxiety, cognitive impairment, and systemic glucose intolerance were shown [245]. These finding demonstrate that the brain requires insulin signaling to maintain its regular functions and the impact of insulin resistance is not only a systemic effect but also directly affects the brain [295].

In a hypometabolic state, amyloidosis increases based on the findings in animal studies and in vitro experiments [296]. Low glucose metabolism and insulin resistance in the brain stimulated BACE1 and GSK-3 activity [297,298]. GSK-3β activation increased APP mRNA expression, BACE activity, tau phosphorylation, Aβ aggregation, and memory impairment, as well as microglia activation-associated inflammatory reactions in AD [298,299]. GSK-3α can modulate APP cleavage and induce Aβ production and that blockade of GSK-3β could prevent Aβ accumulation [300,301]. Overexpression of GSK-3 suppressed LTP by negatively regulating Wnt or PI3K signaling which can lead to memory impairments [302]. GSK-3β also reduced acetylcholine synthesis and induced apoptosis of cholinergic neurons resulting in NFT formation [303].

Insulin accelerates the movement of Aβ from the Golgi network to the plasma membrane, thereby helping Aβ to be released out of neuronal cells [304]. Conversely, Aβ binds to the insulin receptor and inhibits insulin signaling, resulting in LTP inhibition and neuronal spine loss [305]. As a result, insulin suppresses the toxicity caused by Aβ binding to the synaptic insulin receptor [306]. Insulin can also suppress the formation of Aβ oligomer [307]. In addition, insulin has been proposed to regulate extracellular degradation of Aβ by modulating the IDE activity [308]. If the amount of brain insulin decreases, IDE activity also decreases resulting in an increase of Aβ [308]. Additionally, when PI3K/AKT activity is decreased due to decreased insulin signaling, o-glcNAcylation is also decreased, resulting in hyperphosphorylation of tau [309].

Tauopathy is causes cognitive dysfunction by synaptic plasticity disorders and degenerative lesions [310]. In AD tauopathy develops in the brainstem and entorhinal cortex, and its progression to hippocampus and neocortex correlates to the progression of AD symptoms [311]. Tau is also involved in insulin signaling in the brain, and by the lack of tau protein, hippocampal function and the appetite suppression by insulin in the hypothalamus is weakened which results in metabolic impairments [60]. Tau does not interact directly with the insulin receptor and IRS-1 but inactivates PI3K/AkT signaling by inhibiting the activity of PTEN (phosphatase and tensin homologue on chromosome 10), which in turn causes hyperphosphorylation of tau [60,312].

The ApoE4 gene allele is the strongest genetic risk factor for late-onset AD. Insulin signaling was impaired in an age-dependent manner in ApoE-targeted replacement mice and a fatty diet accelerated this impairment [313]. By binding with insulin receptor, apoE4 trapped the insulin receptor inside the endosome and interfered with insulin signaling [313]. This results in decreased mitochondrial respiration and glycolysis, suggesting the role of apoE4 in the pathogenesis in AD in association with insulin resistance.

In summary, decreased glucose metabolism and insulin resistance increase Aβ production by BACE1 activation, deteriorate normal brain functions by the lack of insulin signaling and GSK-3β activation, reduce inhibition of Aβ toxicity and accelerate tau hyperphosphorylation. Additionally, ApoE4 is involved in the insulin signaling process and causes disturbances in energy metabolism.

## 7. Conclusions

AD is a serious social problem, as the prevalence of dementia is gradually increasing in an aging society. However, no proper treatment has been developed yet, and the only effective countermeasure is to prevent the disease progression by early diagnosis. The reason why new drug candidates fail to show therapeutic effects in clinical studies may be due to a misunderstanding of the cause of AD. In this review, hypometabolism and insulin resistance were discussed as one of the main causes of AD.

Insulin resistance refers to a state in which the action of insulin is reduced at physiological insulin concentrations. Insulin has a function of lowering blood glucose concentration to a normal level by introducing glucose into cells through GLUT4. However, insulin also regulates various cell functions. It affects metabolism, survival and differentiation of nerve cells, synaptic activity, and cognitive-emotional function in the brain. Insulin resistance reduces the supply of glucose, which is the main energy source of the brain, affects the metabolism of APP, and causes hyperphosphorylation of tau protein, which can lead to AD. When the main energy source is insufficient, the lipid constituting myelin is consumed, and the volume of white matter in the brain is reduced, which weakens the connections between brain regions. Insulin resistance is pointed out as one of the direct causes of dementia, in that diabetes and AD are so similar that dementia is the so-called type 3 diabetes, and in that glucose metabolism in the brain is reduced 10 years before the onset of dementia symptoms. Additionally, many results have been published indicating that drugs that improve insulin resistance can reduce pathologic changes in AD brain. Therefore, maintaining insulin sensitivity may inhibit the onset and progression of AD.
